# Modulation of Corticospinal Excitability during Action Observation in Patients with Disorders of Consciousness

**DOI:** 10.3390/brainsci14040371

**Published:** 2024-04-11

**Authors:** Mauro Mancuso, Lucia Mencarelli, Laura Abbruzzese, Benedetta Basagni, Pierluigi Zoccolotti, Cristiano Scarselli, Simone Capitani, Francesco Neri, Emiliano Santarnecchi, Simone Rossi

**Affiliations:** 1 Physical and Rehabilitative Medicine Unit, NHS-USL Tuscany South-Est, 58100 Grosseto, Italy; m.mancuso62@gmail.com; 2 Tuscany Rehabilitation Clinic, 52025 Montevarchi, Italy; abbruzzese@crtspa.it (L.A.); pierluigi.zoccolotti@crtspa.it (P.Z.); scarselli@crtspa.it (C.S.); capitanisimone91@gmail.com (S.C.); 3 Dipartimento di Scienze Mediche, Chirurgiche e Neuroscienze, Siena Brain Investigation and Neuromodulation (Si-BIN) Lab, University of Siena, 53100 Siena, Italy; lucia.mencarelli@unifi.it (L.M.); neri.francesco@outlook.com (F.N.); simone.rossi@unisi.it (S.R.); 4 Precision Neuroscience & Neuromodulation Program, Gordon Center for Medical Imaging, Massachusetts General Hospital & Harvard Medical School, Boston, MA 02114, USA; esantarn@bidmc.harvard.edu

**Keywords:** disorders of consciousness, minimally conscious state, vegetative state/unresponsiveness wakefulness syndrome, transcranial magnetic stimulation, action observation

## Abstract

Brain imaging studies have recently provided some evidence in favor of covert cognitive processes that are ongoing in patients with disorders of consciousness (DoC) (e.g., a minimally conscious state and vegetative state/unresponsive wakefulness syndrome) when engaged in passive sensory stimulation or active tasks such as motor imagery. In this exploratory study, we used transcranial magnetic stimulation (TMS) of the motor cortex to assess modulations of corticospinal excitability induced by action observation in eleven patients with DoC. Action observation is known to facilitate corticospinal excitability in healthy subjects, unveiling how the observer’s motor system maps others’ actions onto her/his motor repertoire. Additional stimuli were non-biological motion and acoustic startle stimuli, considering that sudden and loud acoustic stimulation is known to lower corticospinal excitability in healthy subjects. The results indicate that some form of motor resonance is spared in a subset of patients with DoC, with some significant difference between biological and non-biological motion stimuli. However, there was no covariation between corticospinal excitability and the type of DoC diagnosis (i.e., whether diagnosed with VS/UWS or MCS). Similarly, no covariation was detected with clinical changes between admission and discharge in clinical outcome measures. Both motor resonance and the difference between the resonance with biological/non-biological motion discrimination correlated with the amplitude of the N20 somatosensory evoked potentials, following the stimulation of the median nerve at the wrist (i.e., the temporal marker signaling the activation of the contralateral primary somatosensory cortex). Moreover, the startle-evoking stimulus produced an anomalous increase in corticospinal excitability, suggesting a functional dissociation between cortical and subcortical circuits in patients with DoC. Further work is needed to better comprehend the conditions in which corticospinal facilitation occurs and whether and how they may relate to individual clinical parameters.

## 1. Introduction

Disorders of consciousness (DoC) include a spectrum of conditions characterized by different levels of consciousness impairment, which are often secondary to vascular, anoxic, metabolic, or traumatic brain injuries. Because consciousness implies both the level of arousal and the content of consciousness (e.g., functions such as attention, memory, and volition), DoC can independently affect these two domains [1,2]. For example, both arousal and the contents of consciousness are absent in a “coma”. In contrast, the “vegetative state” (VS, better known as unresponsive wakefulness syndrome (UWS) [3]) corresponds to a condition of wakeful unawareness, in which, despite spared sleep-wake cycles, patients show no conscious interaction with the surrounding environment [4]. Further along the spectrum of impaired consciousness, the “minimally conscious state” (MCS) is defined as a condition with minimal and inconsistent—though definite—evidence of awareness, including occasional responses to external stimuli (e.g., making pursuing eye movements tracking the examiner’s finger and the execution of basic verbal commands) [5].

In recent years, several researchers investigated the neural correlates of consciousness [6,7] to identify a neural-based definition of consciousness. Several conflicting theories about the neural correlates of consciousness have been proposed [8] and no conclusions have yet been reached. One of these theories relies on brain complexity as a critical requirement of consciousness [9,10]. According to this hypothesis, consciousness is sustained by patterns of neural activity that are distributed across multiple brain regions and differentiated in space and time. Support for this principle comes from studies using transcranial magnetic stimulation (TMS), coupled with electroencephalographic (EEG) recordings. On the one hand, in patients with VS/UWS, TMS pulses elicit only localized event-related potentials (ERPs). On the other hand, more diffused activity can be elicited in patients with MCS and fully conscious healthy subjects [11,12,13].

Beyond the debate on philosophical and neural theories of consciousness, however, compelling questions with relevant medical and ethical implications remain open when we stand at the bedside of a DoC patient: To what extent can unconscious patients perceive signals from the world surrounding them? Could some kinds of covert, though active, cognitive processing exist in these patients, escaping detection in behavioral and neurological assessments? The definitions provided above, assuming that the contents of consciousness are absent in VS/UWS and are profoundly impaired in MCS, would implicitly rule out clear information processing of the external world, at least in patients with VS.

Over the last few decades, studies employing brain imaging techniques depicted a very different scenario and provided some evidence that residual cognitive processes could be intact in patients with DoC [2]. A recent meta-analysis of studies looking for consciousness-related brain activations in patients with DoC has shown that several cortical areas become activated in some patients when they are engaged in active or passive tasks [14].

Passive paradigms are disparate, including the auditory presentation of isolated words [15] and sentences [16,17,18], self-related stimuli (e.g., listening to one’s name spoken by a familiar voice) [19,20,21], and emotional stimuli (e.g., pain-evoked cries) [22], as well as the visual presentation of complex stimuli such as faces [23]. Overall, these studies demonstrated that residual cognitive processing is detectable at the neural level in some patients with DoC, and the activation patterns are very similar to those observed in healthy subjects [20,21,22,23]. However, brain activation has been often observed to scale with the severity of consciousness impairment (e.g., between VS/UWS and MCS patients) [16,22], suggesting that functional brain imaging might help classify patients with DoC.

Studies employing active paradigms showed even more striking results. A seminal work in this field reported the case of a young woman with VS/UWS who was asked to imagine playing tennis or walking through her house while her brain activity was being recorded using functional magnetic resonance imaging (fMRI) [24]. The pattern of brain activity that resulted was indistinguishable from healthy controls, involving the supplementary motor area when the patient had to imagine playing tennis and the parahippocampal gyrus, the posterior parietal cortex, and the lateral premotor cortex when she was asked to imagine walking through her house. Similarly, when asked to move either their right or left hand, two patients diagnosed with VS/UWS showed functional changes in the contralateral premotor cortex [25]. Notably, the volitional modulation of brain activity during motor imagery is present only in a subset of patients with DoC [26].

In addition to expanding our knowledge of the neural underpinnings of consciousness, studies addressing brain functioning in DoC are crucial to defining reliable prognostic criteria. Currently, the N20 component of the somatosensory evoked potential (SEP) recorded from C3/C4 electrodes after electrical stimulation of the median nerve at the wrist represents the most robust electrophysiological measure for outcome predictions in patients with DoC. Absent or low-amplitude N20 is associated with poor prognosis in patients with DoC, specifically after anoxic injury [27,28,29,30,31,32,33]. However, the presence of N20 is not as effective in predicting a favorable outcome [34,35]. Despite some evidence claiming that spared cognitive ERPs, such as mismatch negativity, could predict favorable outcomes, the results are still quite heterogeneous [35].

Although still seldom employed in this field, TMS is a versatile tool that might help clarify the dynamics of residual cognitive processing in patients with DoC. By delivering TMS pulses over the primary motor cortex, it is possible to probe the excitability of the corticospinal system by measuring the size of electromyographic responses, known as motor evoked potentials (MEPs). Corticospinal excitability reflects the influence of the overall input to the primary motor cortex and the spinal cord [36]. Among many sources of modulation, corticospinal excitability is enhanced during action observation, due to a mirror neuron network involving the frontal and parietal cortices [37]—an effect known as motor resonance [38]. A crucial property of the mirror neuron network is that it shows similar activity when the actions are self-generated and observed in other individuals [37,39]. Individual mirror neurons are often tuned to specific actions (during both action execution and observation) [40] and the pattern of corticospinal excitability facilitation during action observation reflects muscle activity in the observed actions [38]. Mapping observed actions into motor coordinates may underlie our ability to understand and predict what our conspecifics are doing, setting the basis for any form of social interaction. In patients with DoC, motor resonance may, thus, provide a window into the latent processing of social cues that potentially escape clinical observation.

Supporting this concept are the results of a previous study, where facilitation of corticospinal excitability was detected in a subset of patients with VS/UWS who were encouraged to observe and imitate an action presented by the experimenter [41]. Remarkably, those patients showing augmented corticospinal excitability during action observation also improved their consciousness level during follow-up observations after 28 weeks. These results suggest that the assessment of motor resonance in patients with DoC may convey important information for outcome prediction. However, an important limitation of this study was that patients were only presented with intransitive actions (i.e., thumb abduction). Because transitive actions are richer in terms of motor cues, involving a complex relationship between the object and effector, we propose that they would constitute a more suitable approach when targeting covert processes of sensorimotor integration in patients with DoC. In addition, corticospinal facilitation during action observation in these patients might be due to non-specific arousal that is secondary to motion within the visual field, rather than to the specific encoding of goal-directed actions. To clarify this confounding factor, one needs to also assess the effect of non-biological motion observation on corticospinal excitability.

In the present exploratory study, we tested corticospinal excitability in patients with DoC while they were being presented with transitive actions and non-biological motion. In addition, we assessed corticospinal excitability modulation with sudden loud acoustic noises (i.e., the auditory startle paradigm). Like action observation, startle stimuli are known to produce systematic changes in corticospinal excitability (mainly the suppression of excitability [42]). Unlike the mirror neuron system, the neural substrate of the startle response is located subcortically in the brain stem [43]. Corticospinal modulation by cortical (action observation) and subcortical (startle) neural substrates was then correlated with the amount of spared somatosensory input, as indexed by SEPs. Finally, we examined the relationship between the presence of detectable corticospinal excitability and the type of DoC diagnosis (i.e., whether VS/UWS or MCS was diagnosed), as well as possible clinical changes between admission and discharge.

## 2. Materials and Methods

### 2.1. Participants

To be included in the study, patients had to be diagnosed with either VS/UWS or MCS due to vascular or traumatic accidents that had occurred within the previous six months. Patients were classified as VS or MCS based on the JFK Coma Recovery Scale-Revised (CRS-R) and on internationally established criteria [5,44], according to which the emergence from MCS is marked by the detection of a reliable yes-no communication system and/or functional object use. The CRS-R consists of 23 hierarchically organized items, parceled into six subscales designed to assess auditory, visual, motor, language, and arousal functions. Weighted scores are assigned to reflect the presence or absence of specific responses, ranging from brain stem reflexes to cognitively mediated behaviors. The total score can be used to gauge the general trajectory of recovery over time as higher scores reflect progressively increasing levels of cognitive function. The transition from VS/UWS to MCS depends on the scores obtained in the subtests of the auditory, motor, oromotor/verbal, visual, and communication subscales, following the diagnostic criteria set by Giacino et al. [5].

VS/UWS patients fulfilled the following diagnostic criteria: (a) no evidence of awareness of self or the environment and an inability to interact with others; (b) no evidence of sustained, reproducible, purposeful, or voluntary behavioral responses to visual, auditory, tactile, or noxious stimuli, with no evidence of language comprehension or expression; (c) intermittent wakefulness; (d) no presence of epileptic crises; and (e) bowel and bladder incontinence.

The diagnostic criteria for MCS were (a) the ability to follow simple commands; (b) the presence of gestural or verbal yes/no responses (regardless of accuracy); (c) the presence of intelligible verbalization; and (d) the presence of purposeful behavior, including movements or affective behaviors occurring in contingent relation to the relevant environmental stimuli and not due to reflexive activity.

Patients with contraindications to TMS [45] were excluded from the study, as well as those fulfilling the following exclusion criteria: decompressive craniotomy, bilaterally absent brainstem auditory evoked potentials (BAEPs) or visually evoked potentials (VEPs), and the unviability of the corticospinal pathway, as indexed by the absence of motor evoked potentials (MEPs).

Eleven patients (seven females) aged between 46 and 81 years (mean: 70.27 years, SD: 11.21) participated in the study. Six were diagnosed with VS/UWS and five were diagnosed with MCS at the time of inclusion in the study. Demographic and clinical information are reported in Table 1.

The study was conducted according to the Declaration of Helsinki and was approved by the local Ethics Committee of the Siena Health Authority as part of a larger brain stimulation program in VS patients (protocol code: Brainsight; approval number: EME_1144_0_1) [46]. We obtained informed consent from each patient’s legal surrogate.

### 2.2. Clinical Assessment

Patients included in the study were assessed with the Coma Recovery Scale–Revised (CRS-R) during a five-day monitoring period (with one evaluation per day) in the week before they underwent the experimental procedures. The final score was the average over these five evaluations.

The following clinical scales were also carried out to evaluate the outcomes.

*Functional Independence Measure (FIM)* [47]: The FIM is one of the most widely used methods for assessing the basic quality of daily living activities for persons with a disability. It includes 18 items that are designed to determine the amount of assistance required for a person with a disability to perform basic life activities safely and effectively. Each item is rated on a scale of 1–7 (1 requires total assistance, 7 is achieved independently). The activities include a minimum set of skills related to self-care, sphincter control, transfers, locomotion, communication, and social cognition. The range of the FIM score is from 18 to 126 points. Higher scores indicate a greater level of functional independence, while lower scores indicate greater dependence.

*Rancho Level of Cognitive Functioning Scale (LCF)* [48]: The LCF scale is based on observations made by the clinician and is used to assess cognitive functioning in post-coma patients. It was developed for use in the planning of treatment, tracking of recovery, and classifying of outcome levels. It consists of 8 levels for cognitive function, ranging from 1 (no answer) to 8 (the patient is alert and oriented, is able to recall and integrate past and recent events, and is aware of his or her situation).

Finally, in the years following the recordings, we also checked the computerized public health databases to verify the eventual moment of death.

### 2.3. Experimental Procedures

All experimental procedures were carried out at the patient’s bedside. First, we recorded BAEPs, VEPs, and median nerve SEPs; all recordings were carried out following the recommendations of the International Federation of Clinical Neurophysiology (IFCN). Briefly, the BAEPs were recorded by delivering 15 Hz acoustic clicks lasting 0.1 ms. The intensity of the stimuli was 100 dB in the tested ear, while 60 dB of white noise was presented in the contralateral ear. The active electrode was placed on the examined ear’s lobe, while the reference electrode was placed at the Cz location (according to Jasper’s 10/20 system [49]). The ground electrode was placed at the Fz location.

The VEPs were recorded using flashes delivered monocularly through LED goggles. The flashes lasted 5 ms each and were delivered at a 1 Hz frequency. The VEP recording procedure started after 5 min of darkness exposure. The active electrode was positioned 5 cm above the inion on the midline (the Oz electrode, according to the 10/20 Jasper system), while the reference electrode was positioned at Fz.

Finally, upper limb SEPs were recorded from C3 and C4 electrodes using electric stimulation of the median nerve at the wrist (stimulus duration, 0.3 ms; frequency, 3 Hz).

Focal TMS was delivered to the non-lesioned hemisphere using a 70 mm figure-of-eight coil connected to a Magstim 200 stimulator (Magstim Co Ltd., Whitland, UK). In the case of diffuse lesions, the less-affected side was selected after clinical/neuroradiological examination. MEPs were recorded from the first dorsal interosseous (FDI) muscle contralateral to the site of stimulation, using needle electrodes connected to a Neuropack S1 amplifier (Nihon Kohden Corporation, Tokyo, Japan). The sampling frequency was 5000 Hz. The optimal site of stimulation was identified as the point on the scalp where magnetic stimuli elicited the largest MEPs at the lowest stimulation intensity. The resting motor threshold (RMT) was defined as the closest 1% of the stimulator intensity that elicited 5 out of 10 MEPs of at least 50 µV of amplitude [50]. To obtain consistent responses to TMS pulses, during the experiment, stimulation intensity was set at 120% RMT. During all experimental procedures, the coil was oriented with its handle pointing downward and backward, 45° away from the midline.

The experimental design included three conditions: (1) action observation, (2) acoustic startle, and (3) pendulum observation. In patients 1, 2, and 7, the pendulum condition was not run due to technical problems. In the action observation condition, patients observed reaching/grasping actions directed toward objects requiring a precision grip (e.g., a pen, a banknote, or eyeglass temples). Actions were presented at approximately the center of the patient’s visual field and were performed live by one of the experimenters. The TMS pulse was delivered at the end of the reaching phase of the movement, at the point when the experimenter’s fingers started closing on the object. This timing of TMS administration during action observation generates the maximum facilitation of corticospinal excitability in healthy subjects [51]. In the acoustic startle condition, patients heard a sudden and loud bell tone (with a 94 dB Avg/Leq over a 3-second period), which preceded the TMS pulse by 30–60 ms [42]. As conducted previously [42], trials were spaced by at least 20 s to avoid habituation. In the pendulum condition, TMS was delivered while patients were presented with a pendulum oscillating within their visual field at about 2 m from their frontal plane. The TMS pulse was delivered after the pendulum had completed two or three cycles. The pendulum was a wooden sphere of ~3 cm in diameter, attached to a post using a string that allowed it to oscillate. The string length was adjusted to ensure the pendulum oscillated within the patient’s visual field, given that the gaze orientation was often constrained by neck posture. No other individuals were present in the patient’s visual field when the TMS pulse was delivered. One block of 12–16 trials was run for each condition. Block order was randomized across subjects. Two baseline blocks of 12–16 trials were recorded before and after the experimental blocks. During the baseline recordings, TMS pulses were delivered with patients at rest without any external stimulation.

### 2.4. Data Analysis

**Figure 1:**
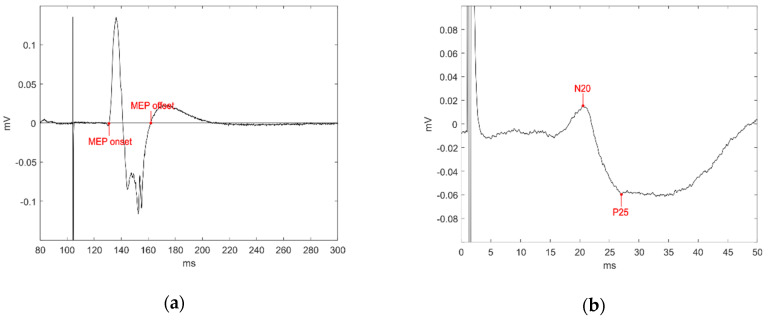
(**a**) An example MEP is plotted. The AUC was calculated between the markers indicating MEP onset and offset; (**b**) the N20 amplitude was calculated between N20 and the subsequent P25 potential.

All the analyses were carried out using custom software written in MATLAB 2018a (Mathworks, Inc., Natick, MA, USA). MEP onsets and offsets were assessed trial-by-trial by visual inspection (Figure 1a) and the area under the curve (AUC) was taken as a measure of corticospinal excitability. Latencies were not considered because only MEPs with the same latency were considered, to be sure that the MEPs’ amplitude modulation reflected the variation of excitability of the same corticospinal pools, according to experimental demands [52,53].

Because the patients included in the study were quite clinically heterogeneous, statistical tests were first performed at the individual subject level. Pairwise independent sample permutation tests based on a t-statistic [54] were performed in each patient to assess significant changes in corticospinal excitability across different conditions. The following comparisons were tested: action observation vs. the baseline, acoustic startle vs. the baseline, pendulum observation vs. the baseline, and action observation vs. pendulum observation. The number of permutations was set at 5000.

For group-level analysis, the average AUC in each condition was normalized by expressing it as a percentage of the baseline AUC. A one-sample permutation test was used to determine the significant changes in corticospinal excitability occurring in each condition compared to the baseline. Due to the limited number of patients, permutation tests were executed with 2048 permutations in the action observation and acoustic startle conditions and with 256 permutations in the pendulum observation condition (i.e., all the possible permutations). Additionally, the ratio between the average AUC during action observation and pendulum observation (AO/pendulum ratio) was calculated in each patient. A one-sample permutation test served to evaluate whether this ratio significantly differed from 1 (i.e., whether action observation determined a significant modulation of corticospinal excitability, compared to pendulum observation).

The baseline-normalized AUC in each condition and AO/pendulum ratios were correlated with the N20 amplitude and the CRS-R scores measures obtained from each patient. As in previous studies, the N20 amplitude was considered to be from its negative peak to the subsequent positive peak (i.e., P25; Figure 1b) [30]. The Spearman rank-order correlation was used for all correlation analyses.

AUC was considered the principal measure of corticospinal excitability. However, peak-to-peak amplitude data were also measured and analyzed to corroborate the conclusions drawn from the AUC data. Peak-to-peak amplitude is a widely used measure of corticospinal excitability in TMS studies [50]. Therefore, its use may help in framing this work within the current and future literature. All the analyses described above for the AUC data were also performed for peak-to-peak amplitude data.

To evaluate their possible relationship with coma severity, both average and normalized AUCs (in action observation vs. baseline and acoustic startle vs. baseline) were correlated with CRS-R scores. Correlations were not examined in the case of the pendulum condition due to a limited number of observations. Similar analyses were carried out on peak-to-peak amplitude data.

Finally, to verify any detectable association with the results of the experimental tests, we examined the individual presence of changes in the FIM and LCF scales between admission and discharge from the rehabilitation ward as a function of individual responses to the experimental manipulations. As a further measure of outcome, we recorded data on the patient’s lifespan. Given the type of data and the limited number of informative patients, these data were analyzed only qualitatively.

## 3. Results

### 3.1. MEPs: AUC

In 8 patients out of 11, the mean AUC values were larger during action observation than at baseline (Table 2). However, this difference was only significant in patients 1, 4, 6, and 11 (*p* = 0.0068, *p* = 0.0040, *p* = 0.0004, and *p* = 0.0010, respectively). In patients 6 and 11, the AUC was also significantly larger during action observation than during pendulum observation (*p* < 0.0001 and *p* = 0.0002, respectively). Moreover, pendulum observation produced a significant increase in AUC compared to the baseline in patients 4 and 10 (*p* = 0.0040 and *p* = 0.0004, respectively). In this latter patient, a significant increase of AUC was also observed in the pendulum condition compared with action observation (*p* = 0.003). The acoustic startle determined a significant increase in AUC compared to baseline in patients 1, 4, 6, 9, and 10 (*p* = 0.0232, *p* = 0.0142, *p* = 0.0104, *p* = 0.0038, and *p* < 0.0001, respectively) and an opposite modulation in patient 11 (*p* = 0.0066).

Table 2 lists the patients as a function of their CRS-R score to analyze the relationship between AUC value in the various conditions and coma severity (and clinical diagnosis). Increased corticospinal excitability, as indexed by AUC, was present in patients with very different CRS-R values and in both patients with VS/UWS and those with MCS. Thus, patient 1, with VS/UWS and a CRS-R score of 1, showed activation for both action observation and acoustic startle (the pendulum condition was not run on this patient); conversely, patients with relatively high CRS-R values (e.g., patient 8) failed to show significant activation compared to baseline, although the direction of the data appears in the expected direction. When examined statistically, the correlations between CRS-R scores and MEP-normalized AUC values in action observation and acoustic startle were negligible and not significant. In the case of average AUC values, there was a marginally significant correlation between baseline AUC values and CRS-R scores (ρ = 0.59, *p* = 0.0544), indicating a tendency for lower AUC values in patients with more severe symptoms. All other correlations were not significant.

At the group level, baseline-normalized corticospinal excitability was facilitated, both during action observation and the startle response, approaching significance in both cases (z = 0.25, *p* = 0.0645; z = 0.48, *p* = 0.0645, respectively). Despite an average increase in MEPs AUC values observed during the pendulum condition compared to the baseline, there were no significant changes in corticospinal excitability with this condition. Similarly, the AO/pendulum ratio was not significant.

### 3.2. MEPs: Peak-to-Peak Amplitude

At the individual patient level, the analysis of MEP peak-to-peak amplitude showed similar results to the AUC data (Table 3). MEP peak-to-peak amplitude was significantly larger during action observation compared to baseline in patients 1, 4, 6, and 11 (*p* = 0.0088, *p* = 0.0032, *p* < 0.0001, and *p* = 0.0006, respectively). In patients 6 and 11, the peak-to-peak amplitude was also larger during action observation compared to pendulum observation (*p* < 0.0001, *p* = 0.0004, respectively). A significant increase in peak-to-peak amplitude was observed in pendulum observation compared to baseline in patients 4 and 10 (*p* = 0.0042, *p* < 0.0001, respectively). Additionally, patient 10 showed significantly higher peak-to-peak amplitude during pendulum observation compared to action observation (*p* = 0.0034). Finally, for the acoustic startle condition, a significant increase in peak-to-peak amplitude was detected in patients 1, 9, 4, 6, and 10 (*p* = 0.0204, *p* = 0.0124, *p* = 0.0074, *p* = 0.0032, and *p* < 0.0001, respectively). In contrast, an opposite modulation occurred in patient 11 (*p* = 0.0078).

In Table 3, the patients are listed as a function of their CRS-R score. No significant correlations were detected between MEP peak-to-peak amplitudes and the CRS-R scores (no ps < 0.10) for either average or normalized values. Furthermore, like in the case of AUCs, the pattern of data for peak-to-peak amplitudes does not show a definite relationship with the clinical diagnosis.

At the group level, only the baseline normalized peak-to-peak amplitude recorded during the acoustic startle condition significantly differed from 1 (z = 0.26; *p* = 0.0391). Furthermore, the corticospinal excitability in the AO/pendulum ratio was also non-significant.

### 3.3. Relationship between the N20 Component and Corticospinal Excitability (AUC and Peak-to-Peak Amplitude)

In at least one hemisphere, all patients showed a detectable and reproducible N20 component. The visual inspection of the scatterplot representing the relationship of baseline-normalized AUC during action observation and N20 amplitude revealed that patient 1 behaved differently from the rest of the sample in this correlation. This patient was, thus, considered an outlier and was excluded from the correlation analysis. After the exclusion of patient 1, the baseline-normalized AUC during action observation was significantly correlated with the N20 amplitude (ρ = 0.83, *p* = 0.0056). Moreover, the correlation of the AO/pendulum ratio with the N20 amplitude approached significance (ρ = 0.69, *p* = 0.0694). Conversely, corticospinal excitability in the acoustic startle and pendulum conditions failed to correlate with the N20 amplitude.

No significant correlations were detected between peak-to-peak amplitude and N20 amplitude in any condition.

### 3.4. Clinical Data at Admission and Discharge

Table 4 reports the clinical data at admission and discharge, as well as the lifespan data of the patients recruited for the study, as were actually available. Small (one-point) improvements in LCF at discharge were present in patients 2 and 7; both patients did not show any significant influence of the experimental conditions on corticospinal excitability (MEPs), as indexed by AUC or peak-to-peak amplitude. A two-point improvement was present in patient 4, who showed increased corticospinal excitability across all three conditions tested (in terms of MEPs AUC and peak-to-peak amplitude).

As for FIM, only patient 4 showed a five-point improvement during recovery, while all other scores were unchanged between admission and discharge.

## 4. Discussion

A general increase in corticospinal excitability during action observation was observed compared to baseline recordings, approaching significance at the group level. Nevertheless, at the individual level, corticospinal excitability was facilitated by action observation compared to the baseline condition in only 4 out of 11 patients (3 of whom were diagnosed with VS/UWS and 1 with MCS). In addition, corticospinal excitability was greater during action observation than during pendulum observation in four (3, 6, 8, and 11) out of the eight patients, although this difference was only significant in two (namely, patient 6, diagnosed with VS/UWS, and patient 11, diagnosed with MCS). However, it should also be observed that three patients (patients 5, 9, and 10) showed the opposite (non-significant) tendency, while one (patient 4) showed equal values. Pendulum observation did not produce a significant increase in corticospinal excitability at the group level and demonstrated no significant difference compared to action observation. These results indicate that, in some patients, corticospinal facilitation during action observation may reflect an intact mirror neuron system operating below the level of consciousness, rather than a non-specific modulation produced by the presence of motion (biological or not) within the visual field. The results on peak-to-peak amplitude generally matched those on AUCs, particularly in the case of individual data.

However, the presence of individual responders in corticospinal excitability modulation did not correlate with coma severity or with clinical diagnosis. There were both responders and non-responders among the VS/UWS patients as well as among the MCS patients. Similarly, changes in clinical scales (FIM and LCF) between admission and discharge were not associated with condition effects on corticospinal excitability. Indeed, only a few patients showed improvements in these scales during recovery; again, these were both responders and non-responders regarding cortical excitability. Notably, FIM and LCF are among the most widely used scales; these have excellent psychometric properties [55,56] but they are still coarse and can identify only macroscopic changes. Finally, when qualitatively examined, there was no clear relationship between the lifespan data and responses in corticospinal excitability.

In a previous study with healthy subjects, a loud and sudden noise suppressed corticospinal excitability when it preceded the TMS pulse by 30–60 ms [42]. Because the optimal auditory stimulus for MEP suppression also produced a strong startle response, previous authors proposed that both these phenomena might arise from the same subcortical neural network, which was likely located in the brainstem [57] and sending upstream projections to the motor cortex. We aimed to study this effect in patients with DoC by presenting a loud bell tone just before TMS delivery. Surprisingly, in our sample, we mostly observed an opposite modulation (i.e., facilitation). As such, a dissociation between cortical and subcortical networks seems to be present in patients who have lost consciousness. However, the results of the current study do not allow us to draw any conclusion about the nature of this dissociation and its clinical relevance. Therefore, further investigations are needed to test this hypothesis.

We failed to detect any correlation between corticospinal excitability and the level of consciousness, as assessed by the CRS-R or performance in clinical scales (FIM and LCF). However, baseline-normalized corticospinal excitability (measured by AUC, although not by MEP amplitude) during action observation (and the AO/pendulum ratio) significantly correlated with the N20 amplitude, with a measure of temporal activation of the primary somatosensory cortex being considered to be of prognostic relevance in DoC [28]. Because we did not carry out a patient follow-up process involving measures of consciousness impairment, our results are inconclusive regarding the prognostic value of corticospinal excitability modulation in DoC (for a recent review, see [58]). However, recent results advocate in this direction [59]. The significant correlation of corticospinal facilitation during action observation with the amplitude of the N20 component suggests (although indirectly) that some predictive information might be extrapolated using this approach. Interestingly, patients with a larger N20 component also showed a larger AO/pendulum ratio, thus being more able to discriminate between human (biological) and non-biological motion. This finding suggests that not only motor resonance per se but also the ability to discriminate between different types of motion might provide valuable prognostic information. Future studies should disentangle the predictive role of information provided by the modulation of corticospinal excitability across different behavioral contexts.

### Limitations

The present, exploratory study had several limitations.

First, one should consider the possibility that the observed changes might have been due to spontaneous fluctuations of corticospinal excitability. This issue could have been dealt with by including resting MEPs in the randomization of experimental conditions. However, this possibility seems unlikely, as nearly all changes in MEP size, when observed, occurred in the direction of experimental predictions.

Second, we did not use a navigation system for coil replacement throughout the experimental conditions. This might have reduced the precision in targeting the motor cortex.

Third, it would have been interesting to correlate measures of corticospinal excitability with MRI findings. However, only CT scans were available for most patients, making this analysis impossible. This remains an interesting target of future research.

Fourth, we did not include a control group of age-matched healthy subjects. However, motor resonance constitutes a robust and reproducible finding in healthy subjects, as confirmed by a large amount of literature published over the last two decades [37,60,61]. Similarly, both the effect of an acoustic startle on corticospinal excitability [43] and the capability of the brain to discriminate biological from non-biological motion were clarified in previous studies [61].

Fifth, the pendulum consisted of a graspable object (a wooden sphere of ~3 cm in diameter). Although the pendulum was out of the range for reaching/grasping movements, visuomotor processing of the sphere affordance might have influenced corticospinal excitability during pendulum observation. To date, the observation of graspable objects modulated corticospinal excitability in healthy subjects [62,63]. Anecdotally, patient 10, an MCS patient and one of the two patients showing corticospinal facilitation during pendulum observation, often exhibited a gesture of attempting to catch the pendulum as she was looking at it. This suggests that, at least in this case, some visuomotor affordance might have been elicited by the mere observation of the sphere. Even though we cannot rule out the possibility that corticospinal excitability might have been affected by pendulum affordance, our results nevertheless support the hypothesis that at least some patients were able to discriminate between pendulum observation and human motion.

Finally, we would like to underscore that the nature of the present study was exploratory. Due to the several strict inclusion/exclusion criteria, obtaining a large sample of patients is cumbersome. Furthermore, patients with disorders of consciousness are notoriously quite variable. These factors contributed to the difficulty of obtaining a sufficiently large sample with, consequently, limited statistical power. Thus, despite our best efforts, the final sample was relatively small, precluding solid statistical group comparisons, and the current results should be considered exploratory. We are currently planning a larger prospective study, which will also take into account the above-described limitations.

## 5. Conclusions

In this exploratory study, we have shown that a certain number of patients with DoC, irrespective of clinical severity, have brain responses to human motion that are similar to those of conscious subjects and differ between the observation of biological and non-biological motion [64]. Conversely, acoustic startle stimuli produced abnormal responses in terms of corticospinal excitability modulation, probably due to a functional dissociation between the cortical and subcortical networks. There was no correlation between corticospinal excitability and the level of consciousness. Similarly, no relationship was detected with the outcome measures commonly used in clinical practice. Whether the modulation of corticospinal excitability may convey prognostic information in patients with DoC remains a fascinating but still unconfirmed possibility. Nevertheless, at least in the case of corticospinal facilitation during action observation, the positive correlation of this measure with the amplitude of the N20 component suggests that the present approach may provide additional prognostic tools in clinical settings. However, further work on larger cohorts of patients is needed to better comprehend the conditions in which corticospinal facilitation occurs and whether and how it may relate to individual clinical characteristics.

## Figures and Tables

**Table 1:** Demographic and clinical information.

No.	Primitive Pathology	Age (Years)	Diagnosis	CRS-R *	Time from Injury (Days)
1	Subarachnoid hemorrhage	76	VS/UWS	1	26
2	Ischemic stroke	59	VS/UWS	5	33
3	Intraparenchymal hemorrhage	72	VS/UWS	5.6	75
4	Subarachnoid hemorrhage	62	VS/UWS	6.4	96
5	Subarachnoid hemorrhage	46	VS/UWS	6.4	60
6	Ischemic stroke	64	VS/UWS	6.8	80
7	Subarachnoid hemorrhage	81	VS/UWS	7.8	121
8	Ischemic stroke	74	MCS	8.4	51
9	Subarachnoid hemorrhage	79	MCS	8.4	130
10	Intraparenchymal hemorrhage	79	MCS	8.4	24
11	Intraparenchymal hemorrhage	81	MCS	9.2	61

* Average of the five-day monitoring period (with one evaluation per day) before the experimental procedures.

**Table 2:** Mean MEPs AUC (mV*ms) across patients and conditions. Patients are listed in the order of increasing CRS-R to appreciate the relationship with DoC severity. Individual data that are significantly different from the baseline are presented in bold (the number in red is a datum indicating an opposite modulation effect). See the text for additional significant comparisons. The standard deviations are shown in brackets.

No.	Baseline	Action Observation	Acoustic Startle	Pendulum	Diagnosis	CRS-R
1	13.58 (6.63)	**28.62 (18.52)**	**25.47 (16.12)**		VS/UWS	1
2	11.18 (2.30)	15.09 (8.28)	13.69 (5.04)		VS/UWS	5
3	20.07 (20.83)	21.54 (18.94)	20.37 (16.48)	19.20 (21.67)	VS/UWS	5.6
4	28.65 (3.29)	**31.32 (1.60)**	**31.01 (2.10)**	**31.32 (1.86)**	VS/UWS	6.4
5	91.82 (20.86)	81.01 (18.40)	95.42 (22.11)	92.00 (17.28)	VS/UWS	6.4
6	20.21 (11.23)	**31.45 (2.92)**	**28.18 (3.14)**	25.84 (4.31)	VS/UWS	6.8
7	43.93 (29.64)	35.66 (23.95)	41.81 (8.91)		VS/UWS	7.8
8	32.63 (12.36)	47.94 (42.04)	46.96 (47.29)	39.80 (26.64)	MCS	8.4
9	26.60 (18.64)	20.15 (8.75)	**46.71 (16.90)**	25.23 (18.84)	MCS	8.4
10	35.90 (24.73)	38.02 (27.13)	**80.32 (44.35)**	**84.18 (46.62)**	MCS	8.4
11	34.16 (24.72)	**59.53 (21.13)**	**15.29 (8.67)**	27.36 (19.30)	MCS	9.2

**Table 3:** Average MEP peak-to-peak amplitude (mV) values across patients and conditions. Patients are listed in the order of increasing CRS-R to appreciate the relationship with DoC severity. Individual data that are significantly different from the baseline are presented in bold (the number in red is a datum indicating an opposite modulation effect). See the text for additional significant comparisons. The standard deviations are shown in brackets.

No.	Baseline	Action Observation	Acoustic Startle	Pendulum	Diagnosis	CRS-R
1	0.38 (0.19)	**0.82 (0.55)**	**0.68 (0.44)**		VS/UWS	1
2	0.50 (0.10)	0.77 (0.38)	0.68 (0.23)		VS/UWS	5
3	1.18 (0.86)	1.20 (0.99)	1.12 (0.80)	1.15 (1.15)	VS/UWS	5.6
4	1.03 (0.11)	**1.11 (0.07)**	**1.10 (0.11)**	**1.13 (0.07)**	VS/UWS	6.4
5	4.15 (0.84)	3.67 (0.82)	3.92 (1.02)	4.51 (0.66)	VS/UWS	6.4
6	0.72 (0.41)	**1.08 (0.16)**	**1.02 (0.10)**	0.93 (0.16)	VS/UWS	6.8
7	1.11 (0.76)	0.98 (0.74)	1.46 (0.63)		VS/UWS	7.8
8	1.08 (0.43)	1.12 (0.40)	1.56 (1.64)	1.32 (0.95)	MCS	8.4
9	0.85 (0.56)	0.61 (0.26)	**1.37 (0.55)**	0.80 (0.55)	MCS	8.4
10	1.42 (0.92)	1.60 (0.91)	**2.83 (1.49)**	**3.09 (1.67)**	MCS	8.4
11	1.44 (0.84)	**2.18 (0.81)**	**0.80 (0.39)**	1.20 (0.81)	MCS	9.2

**Table 4:** Clinical data at admission and discharge and lifespan data. Patients are listed in the order of increasing CRS-R, to appreciate the relationship with DoC severity.

		LCF		FIM		Lifespan (Months)
No.	CRS-R	Admission	Discharge	Admission	Discharge	
1	1	2	-	18	-	1
2	5	2	3	18	18	Alive
3	5.6	2	2	18	18	4
4	6.4	2	4	18	23	41
5	6.4	2	2	18	18	8
6	6.8	2	2	18	18	6
7	7.8	1	2	18	18	35
8	8.4	2	2	18	18	-
9	8.4	2	deceased	18	deceased	4
10	8.4	2	2	18	18	6
11	9.2	3	3	21	-	15

## Data Availability

The dataset is available on request from the authors. The data are not publicly available due to ethical reasons.
